# Monitoring healthy ageing for the next decade: South Korea’s perspective

**DOI:** 10.1093/ageing/afad102

**Published:** 2023-10-30

**Authors:** Eun-Jeong Han, Zee-A Han, Hansuk Kim, Tong Ryoung Jung

**Affiliations:** Health Insurance Research Institute, National Health Insurance Service, Wonju 26465, Republic of Korea; Department of Rehabilitation Medicine, Uijeongbu Eulji Medical Center, College of Medicine, Eulji University, Uijeongbu 11759, Republic of Korea; Division of Disease Control Policy, Ministry of Health and Welfare, Sejong 30133, Republic of Korea; Division of Public Health Emergency Management, Korea Disease Control and Prevention Agency, Cheongju 28159, Republic of Korea

**Keywords:** healthy ageing, monitoring and evaluation, public health, older people

## Abstract

South Korea is the fastest ageing country among OECD countries. Unlike the older generation growing up in the aftermath of the Korean war, the first and second baby boomer generations have heightened expectations regarding public services. In addition to the demand in higher quality of both social and health services by these newer older population, there is a concomitant increased quantitative demand. It is imperative that Korea reimagines their health, social welfare and economic policies to reflect the rapidly changing needs of such generations. One way to do this is to mainstream and continually monitor healthy ageing in all aspects of future policies. In 2021, the Korean Longitudinal Healthy Aging Study was launched in this context, to better understand the needs of the new-older age generation and to produce evidence to support formulation of better tailored policies that could promote healthy ageing. However, Korea is only in its early stage in developing a monitoring system that looks into the performance level of policies that support healthy ageing. As a country that is preparing for such rapid demographic transition and has already commenced developing its healthy ageing indicators, it will be important to assess and monitor uniformly the level of healthy ageing from the framework perspective of WHO. Korea welcomes WHO’s development of an internationally applicable M&E framework for healthy ageing. We hope that WHO’s M&E framework on healthy ageing will help Korea align to the international standards in its journey through the UN Decade of Healthy Ageing 2021–2030 and beyond.

## Key Points

- WHO’s Monitoring & Evaluation framework on Healthy ageing will allow more concrete application of the healthy ageing concept in countries.
- WHO’s M&E Framework will help South Korea to align its healthy ageing indicators to be more internationally comparable.
- South Korea hopes to learn from the Framework and share our experience on the development of national healthy ageing indicators.

## Korea’s demographic trends and the need for a monitoring framework for healthy ageing

Korea is the fastest ageing country among OECD countries. Rates of ageing have accelerated even after 2018 when South Korea became an ‘aged society’ with its older population reaching over 14%. In Korea, older people are defined as 65 and over and it is the standard for qualification for government policies including pension and long-term care services. In 2025, as a result of its first baby boomer generation (people born from 1955 to 1963) turning 65 in 2020, Korea is expected to become a ‘super-aged society’ with more than 20% of its population becoming 65 and over [1]. In addition to this demographic change, in 2033, the second baby boomer generation (people born from 1968 to 1974) will reach 65 and with the addition of these approximately 17 million population, the added on societal and economic impact of ageing in all aspects of Korean society will be enormous.

Unlike the older generation growing up in the aftermath of the Korean war, the first and second baby boomer generations have a high level of education and have experienced much greater economic wellbeing. They have heightened expectations regarding public services and also have a deeper interest in societal, political and cultural issues that affect them.

Furthermore, in addition to the demand in higher quality of both social and health services by these newer older population, there is a concomitant increased quantitative demand. As of 2020, 54.9% of persons aged 65 and over in Korea experienced multimorbidity and the proportion increased with age, from 47.2% for persons aged 65–74 years, 63.3% for those aged 75–84, and 73% for those aged 85 and over [2]. Multimorbidity is a highly associated functioning decline and health-related disability [3, 4, 5, 6, 7] and thus as the proportion of older population grows, people who require both healthcare and LTC services will increase significantly.

Therefore, it is imperative that Korea reimagines their health, social welfare, economic policies to reflect the rapidly changing needs of such generations. Also, Korea must accelerate its transformative approach in viewing older people as not just a vulnerable minority, but as active and valuable members of Korean society. For this approach to be realised, it is crucial that Korea mainstreams and continually monitors healthy ageing in all aspects of future policies in Korea.

## Korea’s efforts to realise effective monitoring of healthy ageing

Korea is only in its early stage in developing a monitoring system that looks into the performance level of policies that support healthy ageing using indicators. Presently, the public health policy in Korea is disease focused and the concept of functioning is not accepted as a main indicator for policy evaluation. Furthermore, there is still no definite consensus among experts on which tool to use to measure the various aspects of healthy ageing. Therefore, it is important to develop a performance indicator and evaluation system that can assess and monitor uniformly the level of healthy ageing from the framework perspective of WHO. Korea already has the Korean Longitudinal Study of Aging (first conducted in 2006) and the Korean Frailty and Aging Cohort Study (launched in 2016) which surveys and tracks the ageing process. However, the surveys have a few limitations regarding under-coverage of the first and second baby boomer generations, insufficient measurements related to healthy ageing, and differences in the purpose of cohort construction.

In 2021, the Korean Longitudinal Healthy Aging Study (KLHAS) was launched in this context, to better understand the needs of the new-older age generation and to produce evidence and base data to support formulation of better tailored policies that could promote healthy ageing [8]. The KLHAS is a large-scale random sample cohort representing people aged 45 years and over in Korea. It is the only nationally representative cohort established based on the concept of healthy ageing in Korea. The KLHAS is made up of the Korean Longitudinal Healthy Ageing Cohort (KLHAC) the Korean Longitudinal Long-Term Care Cohort (KLTC), The KLHAC identifies the causes of intrinsic capacity and functional decline by tracing the trajectories of the ageing process. The KLTC elucidates factors that lead to eligibility into the long-term care system and trace main factors leading to institutionalisation. The baseline study for the KLHAC was conducted from 2021 to 2022, enrolling 10,416 individuals. The KLTC baseline study was conducted from 2022 to 2023, focusing on 5,000 older individuals living in the community who had qualified for LTC insurance. The KLTC also included information on family caregivers and their role in ageing in place(AIP).

The conceptual framework of the KLHAC includes (i) a successful ageing concept model; (ii) a concept to be used for characterisation of frailty syndrome; (iii) a conceptual framework of the ICF; (iv) WHO’s Healthy Ageing concept and UN’s action plan to realize Healthy Ageing for the next decade; and (v) the key performance indicator of the 5^th^ National Health Plan 2030 (HP 2030). The questionnaire of the KLHAC survey was developed and designed with survey tools that are capable of measuring the research details in each survey area based on the conceptual framework. The characteristics of this cohort allows evaluation and surveying of various healthy ageing-related areas, including intrinsic capacity, health behaviours, social support and the environment. Such data can also be linked to various large scale database retained by the National Health Insurance Service (NHIS) that includes medical and long-term care data of the whole Korean population. Such linkages of cohort data and NHIS database can be used not only to further assess and predict the level of contributing factors to healthy ageing but also analyse effective use of medical and long-term care resources for older persons.

The Health Insurance Research Institute is going to launch a study that will conduct the 1^st^ tracing work that will support the development of healthy ageing indicators. Through this study, we expect to provide a policy base to assess and monitor healthy ageing in Korea.

## Timeliness and importance of WHO’s M&E framework

Although the UN Decade of Healthy Ageing outlines important action areas, there is scarce uniform guidance on ways to nationally nor globally monitor or assess healthy ageing. There is also no global consensus on measurement methods for intrinsic capacity nor functional ability, making application difficult in countries.

This is why WHO’s development of an internationally applicable monitoring and evaluation framework is both timely and significant for Korea as well as all countries globally. With WHO’s M&E framework (Framework) and evidence-based recommendations on measures of healthy ageing informed by the systematic review to be published in the special issue, Korea will be able to align its healthy ageing indicators and data collection efforts to be more internationally comparable. Korea welcomes the efforts of WHO and hopes for a two-way synergy where we learn, but also share with the international community our experience and process in developing our cohort and set of indicators that are aligned to the Framework. We are certain that with WHO’s M&E framework on Healthy Ageing, all countries, including Korea, will be able to more concretely apply the concept of healthy ageing into health and LTC policies during the UN Decade of Healthy Ageing 2021–2030 and beyond.
